# Mechanical Response of Fiber-Filled Automotive Body Panels Manufactured with the Ku-Fizz^TM^ Microcellular Injection Molding Process

**DOI:** 10.3390/polym14224916

**Published:** 2022-11-14

**Authors:** Sara Andrea Simon, Jörg Hain, Michael W. Sracic, Hridyesh R. Tewani, Pavana Prabhakar, Tim A. Osswald

**Affiliations:** 1 Polymer Engineering Center, University of Wisconsin-Madison, Madison, WI 53706, USA; 2 Volkswagen AG, Open Hybrid LabFactory, 38440 Wolfsburg, Germany; 3 Department of Engineering Physics, University of Wisconsin-Madison, Madison, WI 53711, USA; 4 Department of Civil and Environmental Engineering, University of Wisconsin-Madison, Madison, WI 53706, USA

**Keywords:** microcellular injection molding, microstructure, fibers, foam, modal analysis, mechanical properties

## Abstract

To maximize the driving range and minimize the associated energy needs and, thus, the number of batteries of electric vehicles, OEMs have adopted lightweight materials, such as long fiber-reinforced thermoplastics, and new processes, such as microcellular injection molding. These components must withstand specific loading conditions that occur during normal operation. Their mechanical response depends on the fiber and foam microstructures, which in turn are defined by the fabrication process. In this work, long fiber thermoplastic door panels were manufactured using the Ku-Fizz^TM^ microcellular injection molding process and were tested for their impact resistance, dynamic properties, and vibration response. Material constants were compared to the properties of unfoamed door panels. The changes in mechanical behavior were explained through the underlying differences in their respective microstructures. The specific storage modulus and specific elastic modulus of foamed components were within 10% of their unfoamed counterparts, while specific absorbed energy was 33% higher for the foamed panel by maintaining the panel’s mass/weight.

## 1. Introduction

**Figure 1:**
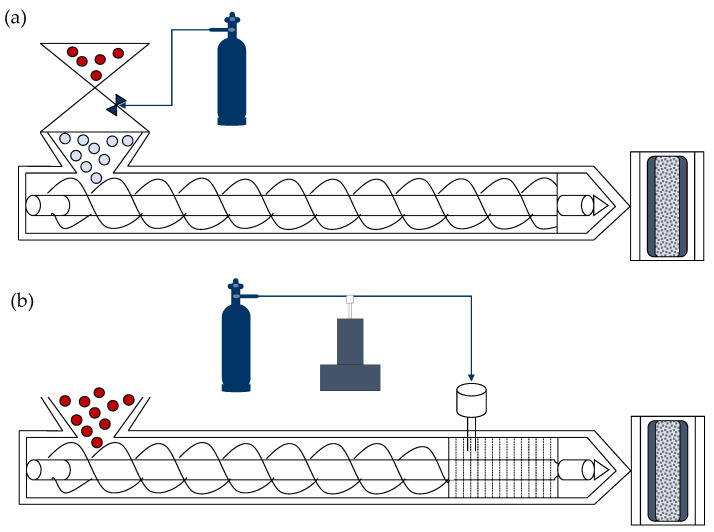
MIM technologies. (**a**) Volkswagen’s Ku-Fizz process with gas supply and a pressurized, modified hopper unit and (**b**) Trexel’s MuCell technology with gas supply, modified screw, SCF metering and control system and a SCF interface kit.

The global demand for battery electric vehicles (BEV) has grown over the past decade to meet climate action targets and governmental energy policies [1]. With a global stock of 16.5 million EVs in 2021 and future market predictions of 200 million EVs in 2030, there is continuous BEV development [2]. To improve driving range and minimize battery size and costs, OEMs have started to investigate new lightweight designs. This study used an alternative microcellular injection molding (MIM) technology called Ku-Fizz in combination with long fiber-reinforced thermoplastics (LFTs) to lighten an automotive door panel. Ku-Fizz is a foaming process which introduces gas with granules at relatively low pressures (<3 MPa) into the feed zone of the injection molding (IM) machine (Figure 1a). As pellets start melting, gas pockets are formed. As the melt continues moving forward in the plasticating unit, these pockets are sheared, compressed, and broken-up until the gas is completely dissolved into the melt. Due to the prolonged exposure time, between gas and polymer along the plasticating unit, sufficient gas diffuses into the polymer, which eliminates the need of additional mixing elements. The injection of the gas-laden melt into the mold cavity induces cell nucleation due to the thermodynamic instability generated by a rapid change in pressure. Cells grow until the melt freezes or until the gas concentration inside the bubbles equals that of the melt [3]. 

Ku-Fizz’s concept and gas pressure operating window (<3 MPa) significantly differs from the leading foaming technology MuCell (<20 MPa). Upgrading traditional IM machines to offer MuCell foaming capabilities involves installing a specially designed screw to generate a single-phase solution and a super critical fluid (SCF) metering, delivery, and dosing system (Figure 1b), while Ku-Fizz uses a modified hopper unit, a standard reciprocating screw, and no SCF system (Figure 1a). A detailed process description can be found in Simon et al., 2021 [4]. 

Both foaming and LFTs have demonstrated their potential in the automotive industry [3,5,6,7,8,9,10,11,12,13,14,15]. Previous work has shown that the foam and fiber microstructure (MS) obtained with Ku-Fizz differs from the leading microcellular foaming technology, MuCell [4]. It was found that the introduction of gas and the presence of foam cells significantly modified the fiber MS. Thus, it is of interest to evaluate how this particular MS impacts the mechanical response of molded components. 

For this purpose, the mechanical response of a SCANIA AB inner truck door panel was evaluated and the difference in response of the foam and compact (=unfoamed, standard density IM part) part was assessed. This door panel is used in all SCANIA trucks and is assembled to the cab door. Several components, such as an electric control unit, a door lock, a door window winder mechanism, and motors, are assembled to the door panel. The principal design attributes for these assembled door bodies are static stiffness, strength, fatigue durability, crashworthiness, and vibration frequency response [16]. The typical door assembly needs to withstand and channel loads into the body of the car. These loads could be horizontal and vertical in nature, such as the force applied in opening or closing doors while leaning/resting on them or when stepping on the armrest to access the roof, or compressive loads during seal compression between the door panel and the steel door [17,18]. These load cases are all highly dependent on panel geometry and mounting.

Door panels also need to withstand a side impact collision to protect the occupant’s chest and pelvis from door contact [19]. During impact, door panels deform, absorb, and disperse energy and transfer impact forces into the surrounding structure [20]. Studies on a Sedan showed doors being one of the highest energy absorbing components (15%) during a side impact event. A total of 66% of the impact energy is dissipated via the bending of the sheet metal of the floor, roof, and door panels [20]. Thus, strengthening the vehicle body-door by using advanced materials and optimizing the design of side impact beams is of high interest to improve the crash behavior [21]. As long fiber composites are increasingly considered for door assembly components, it is imperative to study the material’s dynamic performance under periodic stress to determine the viscoelastic behavior [22]. Dynamic mechanical analysis (DMA) provides information on the process usability of these materials, information on end-user performance, and is key to guaranteeing service performance [23]. 

Door panels respond dynamically to loads, which is also measured in terms of their vibration [24]. Vehicle vibration is a major attribute for customers as they perceive low vibration as increased ride quality, drivability, reliability, and comfort [16,25]. Body panels are designed to avoid resonance with vehicle vibration occurring during normal operation (Table 1) [26]. Vibrations are induced by a number of sources, including road surface conditions, the revolutions of the engine, the brake/suspension system, and the tire imbalance [16,27,28]. For low vibration operation, the first natural frequencies for the bending and torsional modes of body panels must lie within a specific range. These target values depend on the vehicle class and are individually set by OEMs in the initial design stage [16]. For example, the target values for the first bending frequency and the first torsional frequency of BMW three series vehicles were 27 Hz and 30 Hz, respectively (body-in-white) [29]. Bein et al., 2012, reported a first torsional mode target of ~20 Hz for convertibles and >50 Hz for sedans [30]. Other sources revealed a target value for an assembled door of ≥35 Hz for the first inner panel mode. All vibrations under 200 Hz are felt by humans [31].

This study determines the mechanical performance of lightweight foamed SCANIA AV truck door panels and shows its microstructural and mechanical differences to compact panels. First, the fiber and foam MS were measured in key locations to aid in the explanation of the observed mechanical behavior. Second, DMA testing was performed for coupons at various fiber orientations (FO). Third, a side pole impact event was simulated by conducting drop-weight impact tests to determine the energy absorption and evaluate failure behavior characteristics. Fourth, free–free vibration hammer tap tests were performed to obtain the vibration frequency response. The door panel was tested in isolation without any interaction with other components. Testing frequencies were based on loading scenario door panel encounters during normal operation (Table 1).

## 2. Materials and Methods

### 2.1. Material and Manufacturing of Door Panels

The material used in this study is a commercially available 30 wt% long glass fiber-reinforced polypropylene (SABIC STAMAX^TM^). Pultruded pellets have a nominal fiber length (FL) of 15 mm. The fiber diameter was measured to be 19 ± 1 μm using an optical microscope. The material was dried at 80 °C for 4 h to remove any moisture.

SCANIA AB R-Series truck door panels (813 mm × 457 mm × 2.2/2.0 mm) were molded by Idé-Pro on a 1000-ton Demag Ergotech 1000/_1400_-5200 IM machine equipped with a Ku-Fizz hopper unit. The processing settings are summarized in Table 2. The cavity was filled from five gates. 

### 2.2. Measurement of Microstructural Characteristics

**Figure 2:**
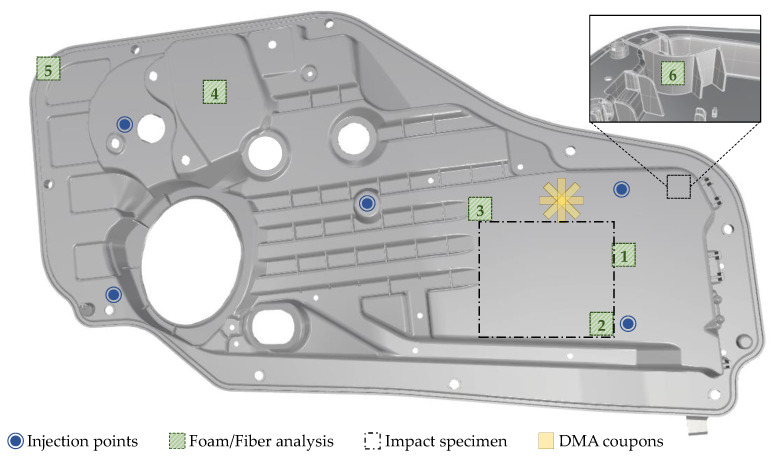
**Investigated door panel.** Sampling locations 1-6 for full fiber microstructure analysis (FL, FC, FO) and foam analysis. DMA test specimen highlighted in yellow, and impact specimen indicated with a black rectangle were extracted from the panel.

The foam morphology and its representative values, CD and CS, were determined by the procedure outlined in [4]. The technique consists of grinding and polishing the embedded specimen, followed by a microscopy and an image processing step. Samples with a cross-sectional area of 40/44 mm^2^ (L × T) were cut out, as shown in Figure 2 (note that compact specimen are slightly thicker).

FO and fiber concentration (FC) were obtained with the X-ray microcomputed tomography (µCT) approach described in [4]. Discs of dimensions 10 mm^2^ × 2.0/2.2 mm were scanned with an industrial μCT system (Metrotom 800, Carl Zeiss AG, Oberkochen, Germany). The resolution of the μCT system is 4 µm. Throughout the reported studies, the voltage was set to 80 kV, the current to 100 µA, the integration time to 1000 ms, the gain to 8, the number of projections to 2200, and the voxel size was set to 5 μm.

The FL measurement procedure presented in [36] was employed in this work. This technique consists of fiber dispersion and a fully automated image processing algorithm to quantify the FL distribution. The Kunc-correction was applied to all results [37]. Sampling locations are shown in Figure 2.

### 2.3. Dynamic Mechanical Analysis

Viscoelastic and damping properties were analyzed with the TA Instruments Rheometric Series RSA 3 DMA equipped with a 35 N load cell. Tests were performed using a 3-point bending configuration with a 40 mm span. Specimens (44 mm × 12.7 mm × 2.06 or 2.15 mm) were extracted at various angles with respect to the flow direction, as shown in Figure 2. As viscoelastic properties are independent of strain at low strain amplitudes, a strain sweep from 0.001–1% at 1 Hz was conducted to identify the linear elastic strain limit. Frequency sweeps from 0.1–100 Hz at a dynamic strain of 0.05% were conducted to obtain damping abilities (tanδ) and storage modulus (E’) for compact and foamed door panels. All tests were performed at room temperature.

### 2.4. Drop-Tower Impact Testing

Rectangular specimens (152.4 mm × 101.6 mm × 2 mm) cut from the door panel (Figure 2) were tested under dynamic impact loading in accordance with ASTM D7136 [38]. Drop-weight impact tests were performed using the INSTRON CEAST 9350 Accelerated Drop Tower Impact System fitted with a 5.72 kg hemispherical striker at the University of Wisconsin-Madison. The impact energy was set to 5 J (1.35 m/s); determined from preliminary tests at various energy values. In total, one compact and three foamed samples were tested. The CEAST DAS 8000 JUNIOR data acquisition system with a sampling rate of 500 kHz recorded force, displacement, and energy versus time responses was used. An anti-rebound mechanism was activated to avoid multiple impacts on the specimens. The Photron FASTCAM Nova S6 high-speed camera with a frame rate of 15,000 fps was used to record sample deformation and assess consequent damage propagation.

### 2.5. Vibration Testing

**Figure 3:**
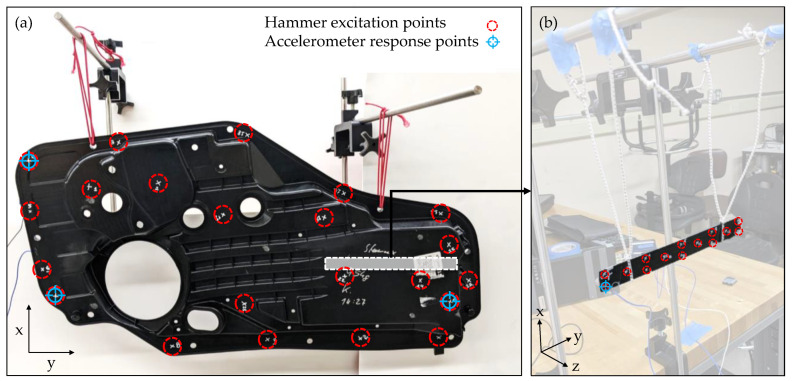
Input and response locations for the hammer tap test. Accelerometers are placed on the backside of parts. (**a**) Door panel. (**b**) Beam extracted from door panel.

To estimate the modal properties of the door panels, a free–free vibration hammer tap test was performed. The panels were hung on an adjustable steel frame with elastic strings as shown in Figure 3a. Three accelerometers (PCB Piezotronics Model 353B15) were adhered to the panels using Petro Wax at edge positions that would allow the detection of bending and torsion in the panel. The accelerometer wire was fixed to the table holding the steel frame. This reduced unwanted noise in the data and minimized the effect of the wire on the stiffness of the elastic suspension of the beam. The part was tapped with a nylon-tipped impact hammer (PCB Piezotronics Model 086C03) on 22 different locations spaced along the door panel, including the accelerometer locations. The locations of the accelerometers and the hammer input locations are shown in Figure 3a.

**Figure 4:**
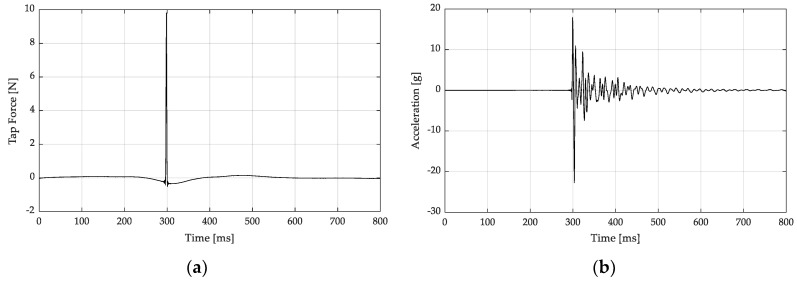
(**a**) Example of the time response of a hammer tap. (**b**) Accelerometer response.

For each hammer input location, the hammer signal and the three-accelerometer signals were recorded with a data acquisition system (Bruel and Kjaer Lan-Xi 3160 A-042 Generator Module). The time domain signals were processed to estimate the frequency response functions (FRF) for each of the three accelerometer locations versus the hammer input location. This was repeated for five hammer taps at each input location, and the resulting FRFs were averaged. Each hammer tap that contributed to the average was verified to ensure the hammer made a single contact with the door panel. Figure 4a shows an example of the measured signal from a hammer input. The sharp peak represents a 9.82 N impulsive force. In Figure 4b, the oscillatory signal is shown with a maximum initial peak which decays to near zero after 0.8 s of response time. 

The algorithm of mode isolation (AMI) was used to determine the natural frequencies and damping ratios of modes in the response [39]. First, the 22 sets of measurements and the associated FRFs were organized into a FRF matrix. The FRF matrix was input to AMI. AMI fits a multi-modal model to a composite of all FRFs. The fit was verified against the FRFs from the measured results to confirm that the best possible fit was achieved.

A modal assurance criterion (*MAC*) analysis was conducted to examine the similarity between compact and foamed mode shapes [40]. The *MAC* was calculated according to Equation (1), where mode j is compared to mode k:

(1) $$MAC_{jk}=\frac{{\left|\phi_{j}^{T}\phi_{k}\right|}^{2}}{\left|\phi_{j}^{T}\phi_{j}\right|\left|\phi_{k}^{T}\phi_{k}\right|}$$

Additionally, the testing of beams was conducted to estimate specific bulk material properties. Beams of 23.0 mm × 25.5 mm × 2.2/2.0 mm were cut from door panels, as shown in Figure 3b (note that the compact specimen is slightly thicker). The testing protocol outlined for door panels was followed. One accelerometer was used. The beam was tapped at 14 different locations uniformly spaced along the beam. FRFs were estimated from the time signals. Five responses were averaged, and modes were fit to the resulting FRFs using AMI. As a first approximation of how microcellular foaming affects the average properties of the composite material, the mode fits were used to estimate the elastic modulus E of the beam samples:

(2) $$E=\frac{\omega_{n}^{2}\rho AL^{4}}{\alpha_{2}^{4}I}$$

where $\omega_{n}$ is the *n*th natural frequency associated with a particular mode shape, ρ is the material density, A is the cross-sectional area of the beam, L is the beam length, $\alpha_{n}$ is the eigenvalue, and I is the cross-sectional second moment of inertia about the z-direction. This estimate was achieved by assuming beams could be represented by a classical Bernoulli–Euler beam model. The development of this model can be found in [41]. The density was calculated to be 1120.02 kg/m^3^ and 1024.1 kg/m^3^ for compact and foamed beams, respectively. 

## 3. Results and Discussion

### 3.1. Foam Microstructure

**Figure 5:**
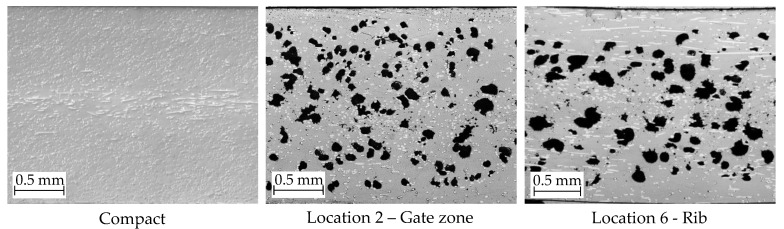
Microscopic images of cross-sections of compact and foamed sampling locations.

**Figure 6:**
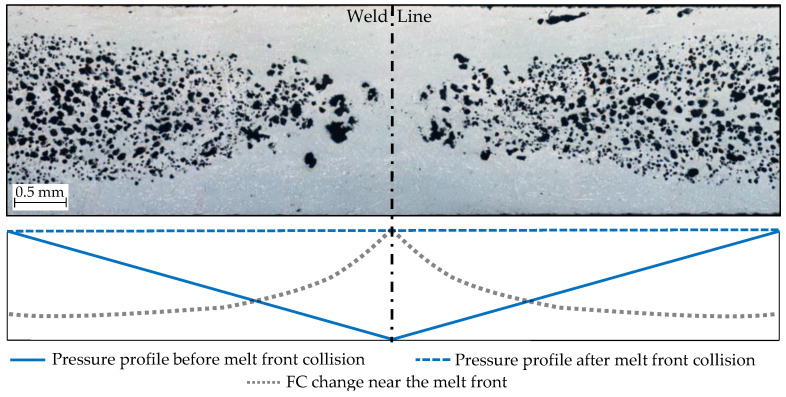
Microscopic image of the weld line at sampling location 1. Schematic of pressure as a function of sample width is demonstrated for before and after melt front collision.

Ku-Fizz produced a cellular structure within the foamed door panels, providing a reduction in material usage and part weight of 90 g or 10%. Figure 5 illustrates the microstructure of compact and foamed specimens. No voids were found for compact panels. A well-defined cell MS was present even in thinner features, such as ribs and insert holders. At the stagnating weld line (Location 1), a reduced CD was observed, which could be caused by a combination of factors. First, the head-on collision of two melt fronts results in a spike of pressure. Cell nucleation and growth could be suppressed or even reversed by this sudden pressure increase (Figure 6). The second FC at the melt front is higher (8%) than the nominal FC [42]. Since fibers are nucleation points, the melt front fills with many small cells which are easily collapsed by the pressure spike.

**Figure 7:**
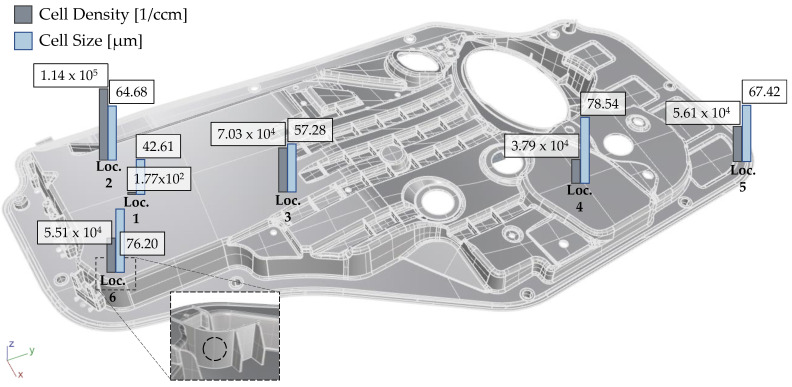
Global characteristic values of foam microstructure.

**Figure 8:**
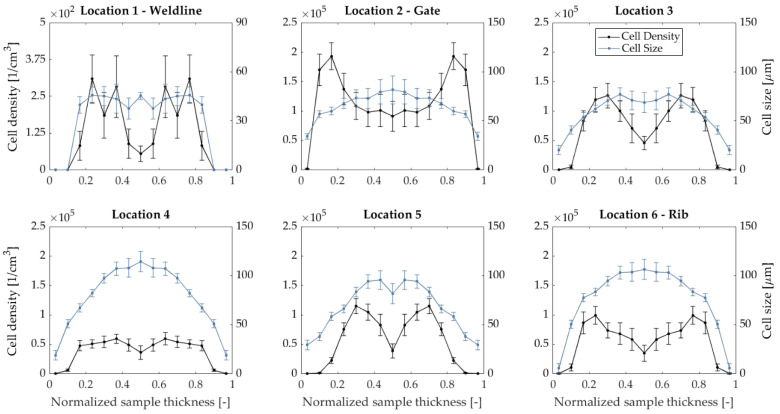
Cell density and cell size across the part thickness for compact and foamed door panels.

Global CD and CS data can be found in Figure 7. Thickness-wise values are shown in Figure 8. CD and CS were plotted as a function of their normalized thickness to regularize the data with respect to variations in sample thickness and to allow a comparison of compact and foamed panels. Values of 0 and 1 represent the surface layer of the molded part, while 0.5 represents the core. CS gradually increases towards the core region [43]. The core has increased melt temperature and reduced matrix viscosity, which allows cells to grow for a prolonged time. As cell growth and cell nucleation are competing mechanisms, and CD drops in the core layer and increases in the shell regions [3,44,45,46]. All samples showed a solid skin layer, which is caused by the rapid solidification of the melt [47,48]. Similar to fiber microstructure, CD and CS distributions impact the mechanical response of foamed components [49]. 

### 3.2. Fiber Microstructure

**Figure 9:**
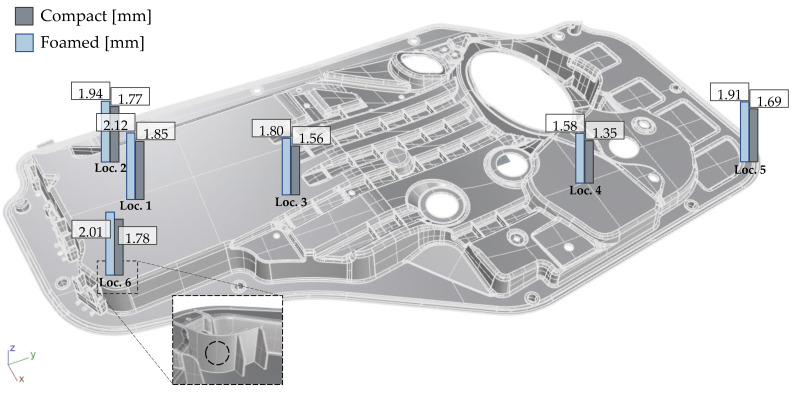
Global characteristic values L_W_ of fiber microstructure.

**Figure 10:**
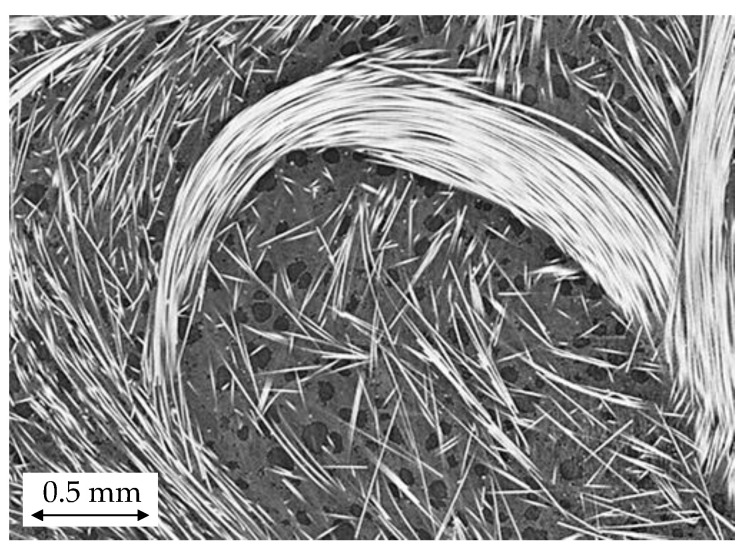
Undispersed fiber bundle in foamed door panel at location 3. Dark area indicates cells, gray area represents the polymer matrix, and fibers are shown in white.

As shown in Figure 9 and previous work [4,50], the processing of glass fiber-reinforced PP in the presence of gas reduces the occurrence of fiber breakage during processing. For foamed and compact panels, a weight average length, L_W_, of 1.89mm and 1.67 mm were recorded, resulting in a 14% increase in FL. While L_W_ values significantly differ, a number average length, L_N_, of 0.82 mm was obtained for both compact and foamed parts; this implies that very long fibers were preserved in the foamed panels. Dissolved gas acts as a plasticizer, reduces melt viscosity, and thus reduces fiber attrition [50]. Largest L_W_ values were recorded at the gate and at the edges of the panels (Figure 9). Close to the gate, fibers experienced shorter flow length and, therefore, have experienced a gentler stress history, leading to an increased L_W_. As shown by Bechara, 2021, longer fibers tend to accumulate at the melt front and are deposited where the melt front stops. Fibers travelling in the melt front come from the core layer where stresses are low and, thus, damage is reduced [51]. Increased FL at the end of the flow path was also noted by Goris and Phelps et al. [42,52]. Partially undispersed fiber bundles were observed in compact and foamed panels (Figure 10). Since dissolved gas acts as a plasticizer, it would be expected that foamed parts have poorer dispersion and are more undispersed fiber bundles. However, no significant difference was observed between foamed and compact parts. Intact fiber bundles act as stress concentrators and result in aesthetic problems. 

**Figure 11:**
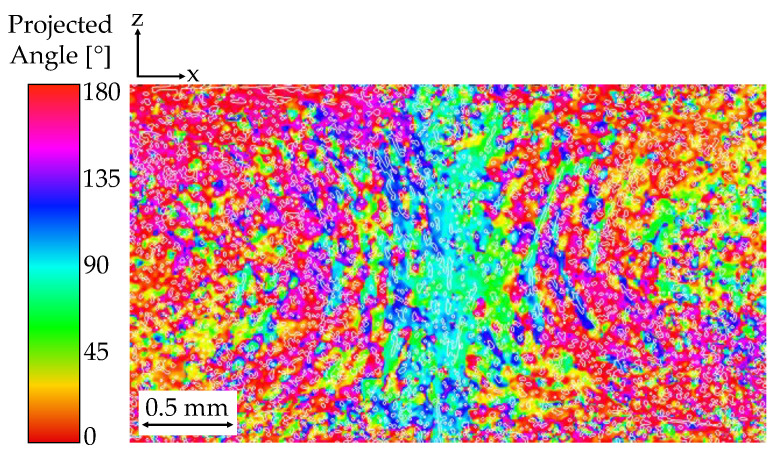
Fiber orientation gradient approaching the weld line (location 1). Blue/turquoise colors indicate increased FO in A_YY_. Red/pink fibers show alignment in A_XX_.

**Figure 12:**
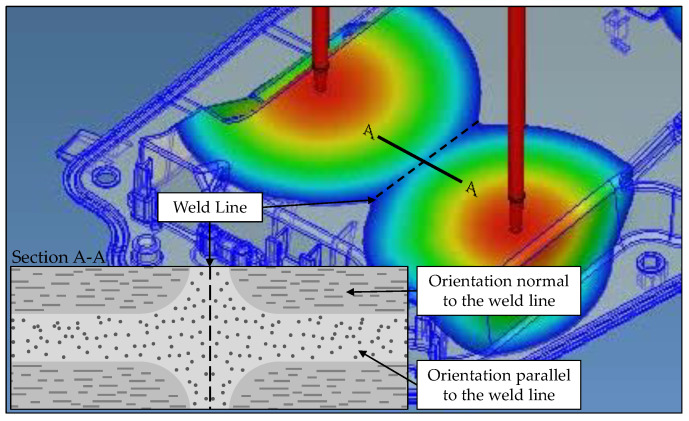
Schematic representation of the FO at a stagnating weld line. Moldex3D mold filling simulation was conducted by SimpaTec Inc. (Aachen, Germany).

The presence of gas changes the matrix rheology, effectively modifying the velocity profile and its effect on FO. Additionally, bubble growth displaces and re-aligns fibers [4,50,53]. The global FO for compact and foamed panels is shown in Table 3. Close to the gate, FO is mostly random due to the shorter flow length and the limited time for fiber alignment under the radial flow inside the cavity. Fibers gradually become aligned perpendicularly to the flow direction (A_XX_) as the flow distance increases [54]. Location 1 represents a weld line of about 0.5 mm in width, extending throughout the part thickness. It can be seen in Figure 11 and Figure 12 that fibers at the weld line are predominantly oriented parallel (A_YY_) to the weld line [55]. 

Foamed panels presented slightly less alignment in the flow direction when compared to their compact counterparts. These findings agree with published studies. Kim et al., 2019, found that cell growth induced the randomization of the FO in MuCell foamed glass fiber-reinforced PP plates [56]. Yang et al., 2022, noted that foamed specimens exhibited a lower fiber alignment along the flow direction when compared to solid samples [57]. Ameli et al., 2014, also showed attenuated preferential fiber orientation [58]. While these authors studied thin-walled parts (≤3.2 mm), Sykutera et al. found that thick-walled parts (>8 mm) do not exhibit any reduction in fiber alignment [59]. As FO is an important variable for fibrous composites, these changes in fiber alignment will impact the mechanical properties of the door panel.

**Figure 13:**
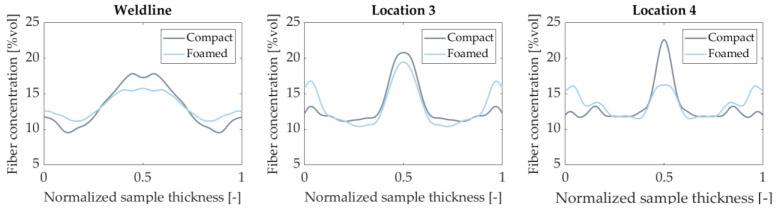
Symmetrical fiber volume fraction for compact and foamed panels at various sampling locations.

Cell growth in the core region tends to displace fibers towards the mold surface, effectively changing the FC profile [4]. This phenomenon was observed for all sampled locations. Figure 13 shows how foaming impacts FC at three different locations. Even at the weld line, where cell growth is limited, a slight reduction of FC in the core is observed. Thickness wise fiber migration is an important effect as it directly impacts bending stiffness [42,60,61]. 

### 3.3. Dynamic Mechanical Properties

**Figure 14:**
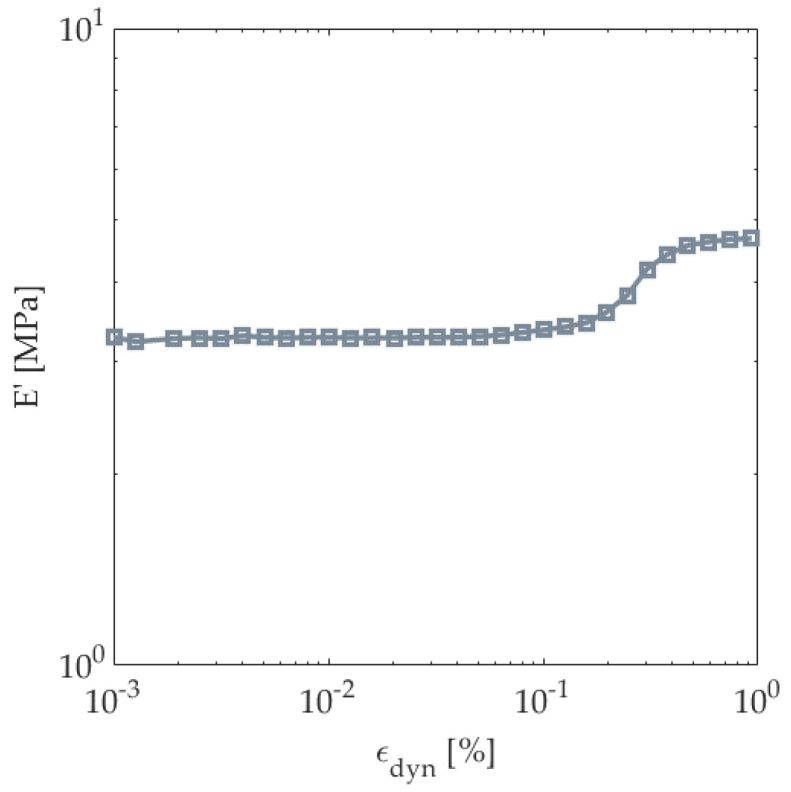
Strain sweeps at constant frequency for composite door panels.

The behavior experienced during the dynamic strain sweep can be seen in Figure 14. The storage modulus (E’) remains constant until a dynamic strain limit of 0.05% is reached. Above this value, the collapsing of cells and nonlinearity of the matrix can occur. 

**Figure 15:**
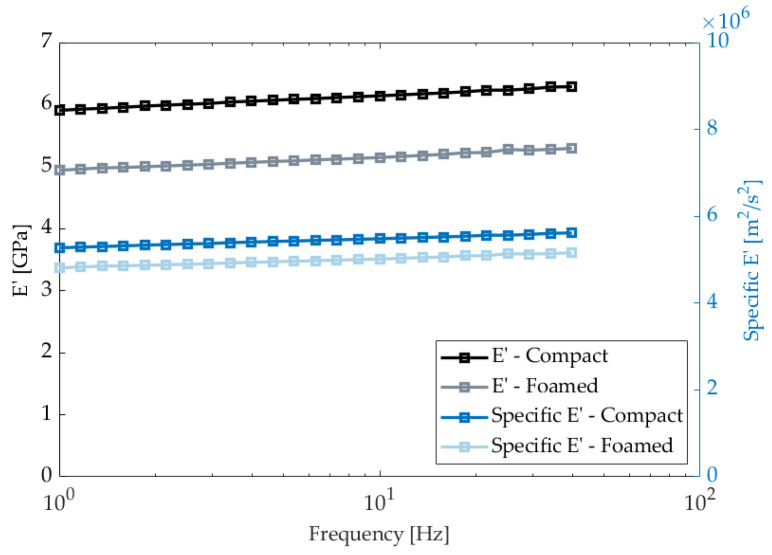
Average storage moduli and specific storage moduli for compact and foamed panels.

To study the difference in E’ between compact and foamed panels, coupons were cut in various orientations with respect to the flow direction to randomize the effect of FO. Measurements were taken until 46 Hz, as samples would lose contact with the bending fixture. Results from the isothermal frequency sweeps provide valuable insight into the behavior of door specimens at different time scales (Figure 15). E’ increases with frequency as the material appears stiffer. An average E’ of 5.99 GPa and 5.02 GPa was determined for compact and foamed specimens, respectively. Unfoamed panels showed a 20% higher E’ over the tested frequency range. However, when comparing the specific storage moduli, this gap was reduced to 9%. This finding highlights that foamed panels perform similarly per unit of weight, making Ku-Fizz a good alternative to lighten automotive door components.

Average tanδ values of 0.0391 and 0.0394 were found for foamed and compact conditions, respectively, over the tested frequency range. The presence of cells does not significantly impact material damping. This can be attributed to the lack of cells near the coupon surface; the region that carries most of the bending stress. tanδ values showed little variation along the tested frequencies as glass fibers reduce tanδ’s frequency dependence. These results align with previous DMA studies conducted on long glass-fiber reinforced PP [62]. 

### 3.4. Mechanical Response from Impact Tests

**Figure 16:**
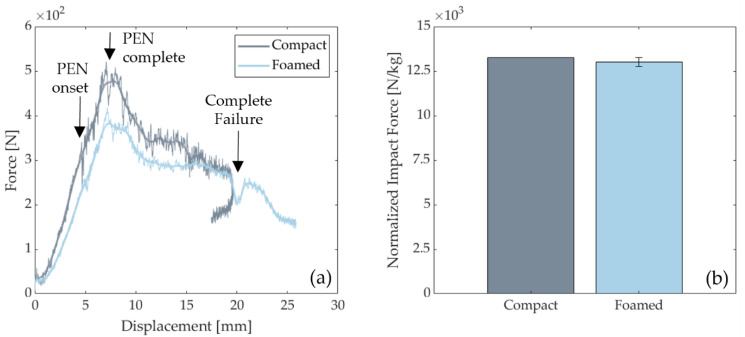
(**a**) Force-displacement plots of original and smoothed for compact and foamed specimens. PEN indicates penetration. (**b**) Impact force normalized with sample weight.

Figure 16a shows the representative force-displacement response of compact and foamed specimens. Based on the force-displacement responses, the equivalent impact stiffness was 222 N/mm and 310 N/mm for foamed and compact parts, respectively. The compact specimen exhibited a sharper drop in the post-peak regime, which indicates higher damage as higher energy is dissipated over a short global deformation (Figure 16a). Open curves without rebound can be seen for all tests, indicating the complete penetration and perforation of both types of samples [63,64].

Figure 16b shows an increased impact force for a compact specimen as air bubbles near the surface cause foamed parts to fail more easily [65]. To account for the difference in densities, the impact force was normalized with the respective sample weight. The gap in impact force reduced from 20% to 2% when values are normalized (Table 4). Thus, foamed door panels can compete with compact ones for weight-sensitive parts. 

**Figure 17:**
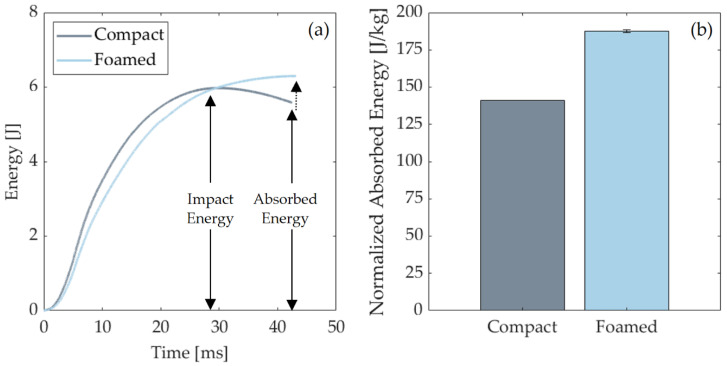
(**a**) Energy vs. time output. (**b**) Normalized absorbed energy for 5 J energy levels. Samples were normalized to their respective weights.

Both compact and foamed specimens absorb impact energy by a complete failure. Figure 17a shows the energy-time graphs, where absorbed and impact energies are indicated. Figure 17b presents the weighted normalized absorbed energy. Foamed specimens absorbed 33% more energy prior to complete failure than compact samples (Table 4). This is partially explained by the presence of air bubbles in the foam core, which act as dampeners. Additionally, the introduction of gas modifies the fiber MS, which also influences impact response. This explains the different energy/time curve types in Figure 17a as the foamed MS has the capacity to absorb higher energy per unit of mass. 

**Figure 18:**
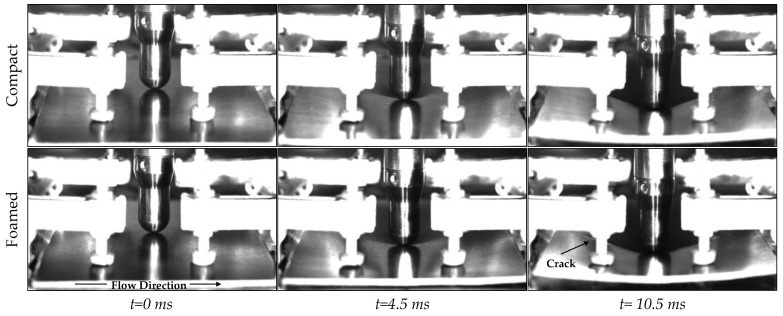
High speed camera images showing the impact event, the bending, and the deformation and fracture of a compact and foamed specimen.

**Figure 19:**
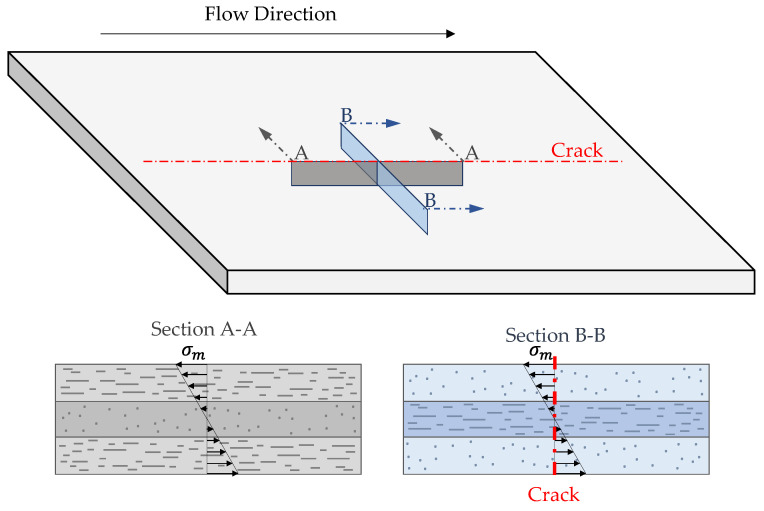
Anisotropic response of impact specimens.

Figure 18 shows the stages of the impact test. Upon impact, one can see elastic deformation (indentation and elastic bending), which is followed by the formation of cracks and ultimately complete penetration. As seen in the last frame for the foamed sample (t = 10.5 ms), the crack propagates in the same direction as the flow path during mold filling. Since fibers are predominantly aligned with the flow, the sample is more resistant to failure due to bending in this direction [42]. Figure 19 illustrates the anisotropic microstructure and how fibers in plane B-B are normal to the load direction in the outer regions where stresses are highest. In this plane (B-B), the matrix will carry most of the load near the part surface leading to an earlier failure, most likely due to debonding. This mode of failure is known as transverse cracking [66,67].

**Figure 20:**
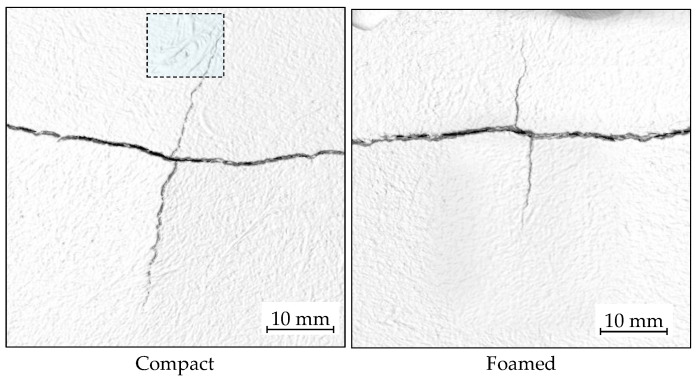
Transversal crack propagation on the back surface for compact and foamed samples. The high-lighted area shows a high concentration of fibers due to undispersed fiber bundles.

Crack propagation was evaluated using micro-CT scans. Figure 20 shows examples of the secondary cracks formed perpendicular to the main crack direction. The presence of the foam core can have different effects on the crack propagation behavior due to hindrance caused by air cells. This can force the crack to undergo a tortuous path, which reduces the extent of crack propagation. In some cases, the crack can deviate from a straight path by following a weaker path generated by large cells and changes in fiber density [68]. For example, Figure 20 (left) shows the upper crack avoiding a high FC region caused by undispersed fiber bundles. In other cases, cells can relieve the high stresses at the tip of the crack and stop crack propagation [69,70]. 

Table 5 presents the length of transverse cracks. It can be seen that voids reduce transversal crack propagation by 50% on the impact plane and 38% on the part’s back side. This highlights how cells can reduce crack propagation in a similar way, as drilled holes prevent crack growth in wind shields or aerospace panels [71].

### 3.5. Vibration Testing—Modal Analysis

**Figure 21:**
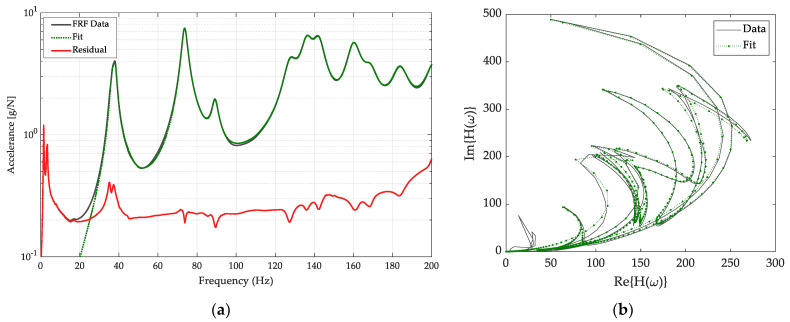
(**a**) Compact panel FRFs. The dark gray line indicates experimental FRF data, the green dotted line represents the AMI fit and the red line shows the residual (error) after refinement of a multi-mode model fit. (**b**) Complex plane plot of composite. H(w) is the FRF.

Hammer-tap tests were conducted to determine the effect of MIM on the vibration response. Natural frequencies from the modal analysis of acceleration FRFs were estimated by fitting prominent peaks in the composite FRF with AMI. The first two peaks in the range of zero to five Hz were not fitted, as these modes were identified as rigid body modes of the panels on the suspension system and were omitted. Following all the individual fits, AMI refines a multi-mode model that includes all the individually fit peaks. Figure 21a shows an example of the AMI fit for a compact door panel. The composite of all 66 FRFs is shown in the solid line (FRF Data), the fit of the composite of the multi-mode fit (Fit) is represented by the dashed line, and the difference between the FRF Data and the Fit (Residual) is shown by the solid red line. The residual is reduced from the FRF data by an order of magnitude for most of the frequency range, thus the fit matches the composite FRF curve well. Figure 21b shows the complex plane plot, also known as the Nyquist plot of the FRF. The peak with the highest magnitude in Figure 21a, corresponds to the crescent furthest to the right in Figure 21b. 

**Figure 22:**
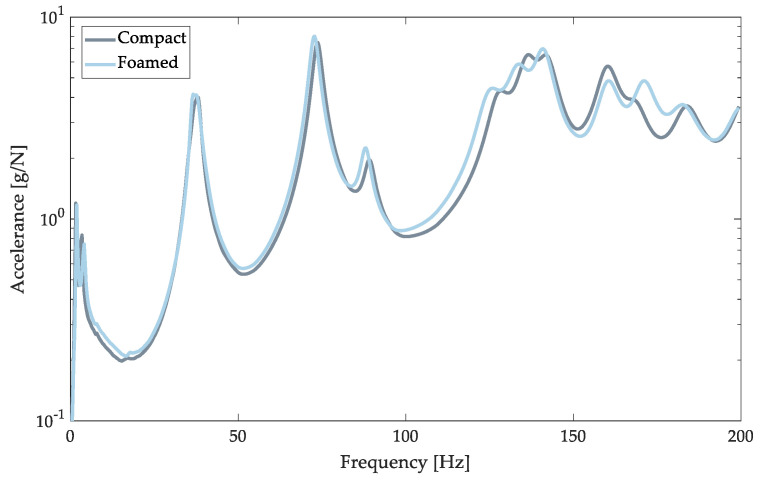
Composite FRF of compact (solid gray) and foamed (solid blue) door panels.

The composite FRF for the compact and the foamed door panels is shown in Figure 22. The natural frequencies and damping ratios were extracted from the AMI results (Table 6). Eleven modes were detected for both panels with frequencies that ranged from 36 Hz to 200 Hz, however, only the first nine modes were reliable (low noise). The modes of the foamed panel are shifted in their locations of peaks and heights of peaks. Generally, the presence of gas in the MIM process shifted modes to lower frequencies as the static modulus decreases, the material becomes softer, and thus frequency decreases [72]. 

Regarding the damping of composite plates, it can be noted that compact door panels exhibited better damping properties for modes 1, 3, 4, and 7–9 than their microcellular counterparts. This phenomenon could be explained by the presence of localized large voids introduced by the dynamic nature of MIM [73]. In addition, Suarez et al., 1985, reported increased damping characteristics for composites with low fiber aspect ratios [74]. In this work, compact panels showed reduced FL when compared to their foamed counterparts. For modes 2, 5, and 6, increased damping ratios were recorded for foamed parts. The presence of gas increases the crystallinity of the material, which in turn can increase damping properties [75,76,77]. Additionally, gas reduces the matrix viscosity, which alters FO and FC, and thus affects frequency and mode characteristics [4,78,79]. Bozkurt et al., 2016, found that the damping characteristics of basalt/epoxy composite laminates strongly depend on FO [80]. In general, material damping in fiber-reinforced composites is a complex phenomenon that causes difficulties in obtaining accurate damping ratios. This is likely caused by measurement errors that are large in comparison to the FRF amplitudes, thus making quantification difficult.

**Figure 23:**
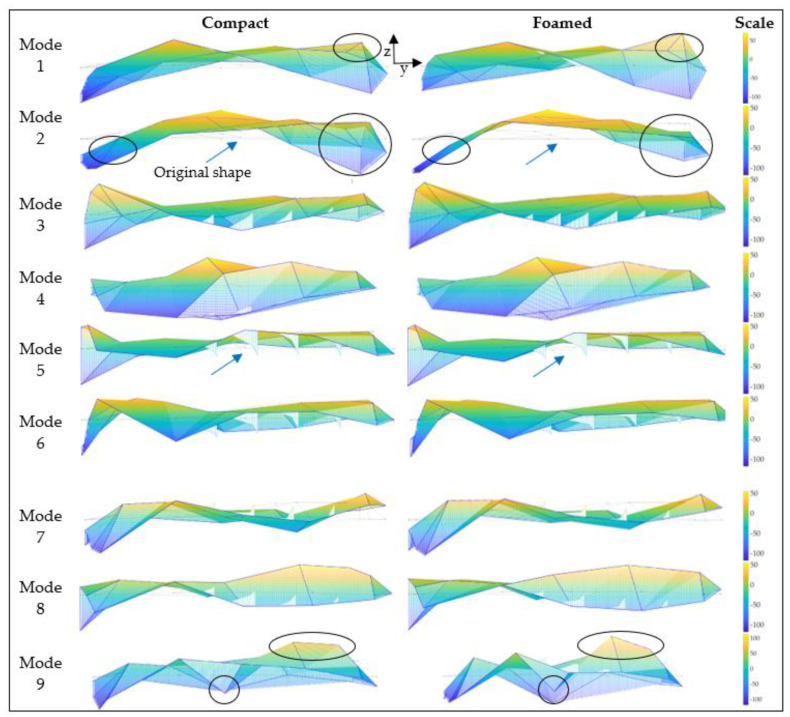
Plate mode shapes for compact and foamed door panels. Dotted lines represent the undeformed wireframe of the panel (indicated by arrows). Circles highlight local differences in deflection. Lined triangles jutting from the part are caused by the employed surface color visualization method.

**Figure 24:**
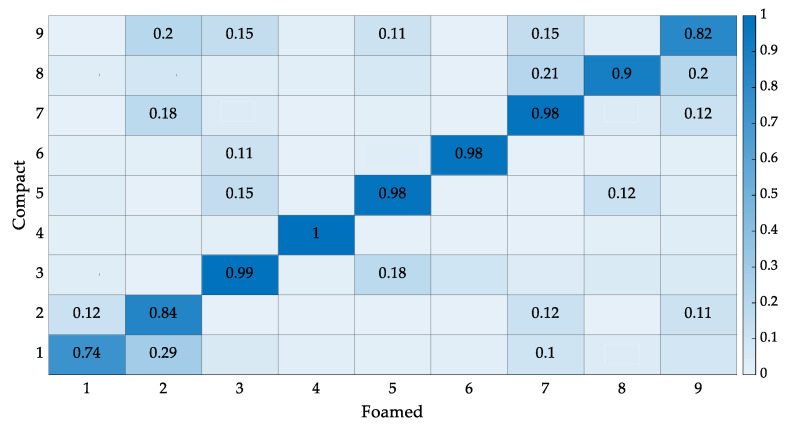
MAC matrix. Mode shapes above a MAC value of 0.1 are displayed.

Plate mode shapes for compact and foamed panels are shown in Figure 23. Their corresponding mode descriptions based on visual inspection, as can be seen in Table 6. Modes are labeled as plate modes if they display both bending and torsion prominently, where corners opposite each other on a diagonal across the panel deflect with the same phase but are out of phase with the nearest corner on an edge. Modes are labeled as bending modes if the shape is dominated by bending and exhibits minimal torsion. Analyzing Figure 23, similarities between mode shapes are evident, with local differences in deflections. For example, mode 1 for the compact panel deflects more relative to the rest of the panel when compared to its foamed counterpart. More differences can be noted. However, quantifying these variations visually proves difficult as door panels are large, complex specimens with complicated features and cutouts. To study the similarities and differences between modes quantitively, a MAC analysis was performed (Figure 24). If modes are identical, the orthogonality properties of modes yield a value of 1. If mode shapes differ, a low MAC value is attained. Typically, diagonal values greater than 0.95 and off-diagonal terms less than 0.1 are desired to confirm mode shape alignment. Figure 24 shows four diagonal values less than 0.95, with the lowest diagonal term being 0.74. There are 17 off-diagonals greater than 0.1, with the largest off-diagonal term of 0.29. Modes 1, 2, 8, and 9 show differences between compact and foamed door panels. Modes 2, 8, and 9 are primarily bending modes. Variations in these modes indicate that the MS of foamed panels affect bending strains more significantly than torsional strains. 

It is important to note that the composition and structure of the object, material anisotropy, part density, L/T ratios, and the manufacturing process all influence the vibration response at various degrees, and thus, changes in modes and their shapes cannot be reduced to one physical characteristic. Vibration response characteristics and thus mode shapes are affected by cell generation, which in turn affects the fiber microstructure. This work has shown that the presence of gas results in an increase of FL, modifies the FC profile, and increases normal to flow fiber alignment due to reduced matrix viscosity. Longer fibers and higher fiber volume fractions increase the stiffness of foamed panels and, therefore, increase natural frequencies [80]. During injection, cell nucleation and bubble growth cause fiber migration towards mold walls, leading to door panels with stiffer shells; simultaneously, the presence of a foam core reduces stiffness. Additionally, mode shapes vary with physical characteristics, such as density and part thickness [81]. Part mass decreased by 10% and average part thickness decreased by 6% when using MIM. As shown by Talekar et al., 2020, and Shishir et al., 2022, for bending, as part thickness decreases, natural frequencies decrease as well [82,83]. This decrease was also observed in the present study.

**Figure 25:**
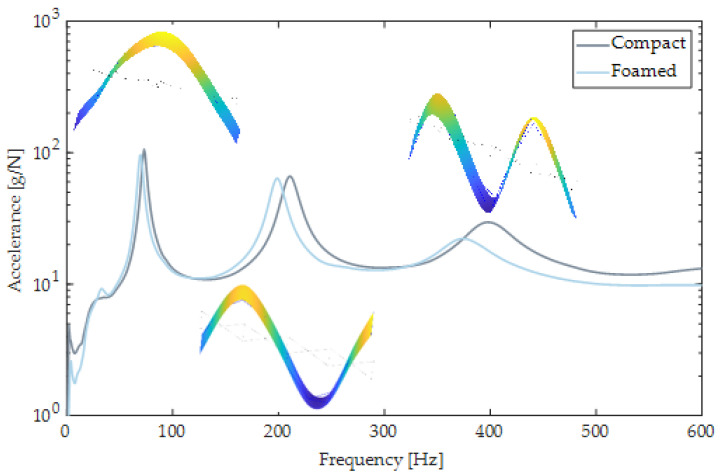
Composite FRF of compact (solid gray) and foamed (solid blue) beams. Mode shapes are shown for the foamed beam cut-out.

Beam cutout samples were also used to reveal differences between foamed and compact door panels. The frequency span was limited to the first three natural frequencies, and the corresponding composite FRFs are shown in Figure 25. Three distinct peaks can be seen, ranging from about 70 to 600 Hz. Their mode shapes were inspected and consisted of the Bernoulli–Euler beam bending modes 1, 2, and 3. Their frequency values and damping ratios are listed in Table 7. For foamed panels, peaks are shifted to lower frequencies by about 6% for each mode. Damping ratios in the foamed beam increased by about 8% for the first and third modes and was reduced by 2% for the second mode. 

The estimated elastic modulus is shown in Table 8. Elastic moduli ranging from 3.28–3.93 GPa were observed. A decreased modulus of about 8% was noted for foamed beams when compared to their compact counterparts. This indicates that for bending strains, the microstructure of foamed panels reduces the panel stiffness when using a first approximation to the anisotropic structure. This decrease in modulus is caused by the reduced density of foamed beams as well as the reduced natural frequencies. As door panels are interior automotive components, they are required to have a high stiffness to weight ratio. Foaming improves specific mechanical properties. The estimated specific modulus (E/ρ) slightly increased by 1% for the foamed beam specimen. For a more accurate modulus determination, a smaller impact hammer should be used to reduce noise in vibration responses. Additionally, a model accounting for the anisotropic behavior of composite beams will yield a more accurate representation of the material behavior.

## 4. Conclusions

The MS resulting from the Ku-Fizz process caused relevant changes in the mechanical response of door panels. The introduction of gas affects structural variables in two ways; first, it generates a cell distribution; second, it modifies the matrix rheology; and thus, it impacts the main fiber characteristics (FL, FC, FO). For foamed panels, a 14% increase in FL (L_W_), increased randomization of FO, and fiber displacement towards the mold surface were observed. Cell MS analysis revealed high CDs and small CSs close to the cavity wall and decreased CD and larger cells in the core region of the part. CD values up to 1.14 × 10^5^ were recorded. The impact of gas on the MS varies in magnitude and is dependent on the gas pressure operating window, which is one of the main differences between traditional MIM Mu-Cell and the more recent technology Ku-Fizz. 

For DMA testing, a 3-point bend configuration and 0.05% strain were used. For these conditions, compact samples showed a 20% higher E’ over a 1–50 Hz frequency range. tanδ exhibited little difference between foam and compact specimen. Modal analysis revealed a shift towards lower frequencies for MIM panels. The general shape of the vibration modes presented little change. The modulus determined from the beam cutout sections decreased for foamed samples for the first three modes of vibration. Unexpectedly, the presence of foam cells increased the absorbed energy before reaching a complete failure during impact testing. Cells act as stress relievers, slowing down or hindering crack propagation in the transverse direction. 

Ku-Fizz is a cost-effective competitor to the leading MIM technology, MuCell. Upgrading an IM machine to offer Ku-Fizz foaming capabilities requires an upfront capital investment of USD 40–60,000 depending on the IM machine size. In comparison, the upgrade costs for MuCell are approximately USD 180–300,000 or 35% of the IM machine cost [84]. The additional economic benefits of Ku-Fizz stem from lower gas pressures needed (<3 MPa), the possibility to install the hopper unit on any IM machine, the option to run compact molding trials by opening the chambers in the hopper unit and avoiding the need to alter the reciprocating screw. As an example, Ku-Fizz was employed to lighten a glass fiber PP+EPDM floor panel of a SEAT Leon in a serial application. Results showed a part weight reduction of 11% due to foaming and a cost reduction of 12% or USD 0.12 per part [8]. 

**Figure 26:**
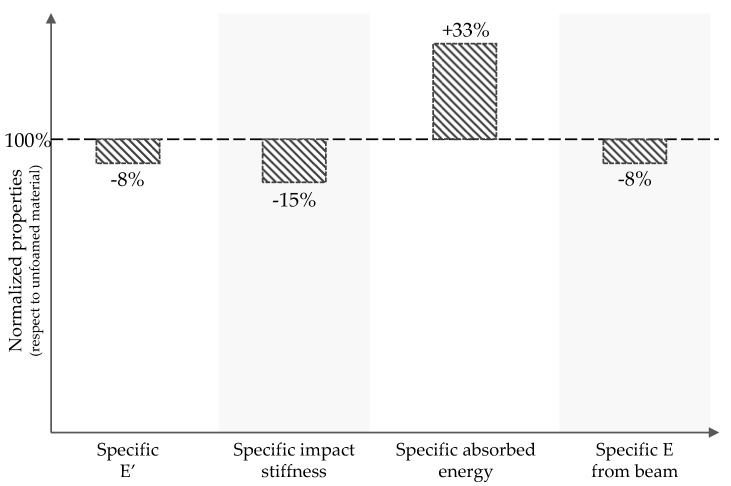
Normalized mechanical properties with respect to compact specimen.

As vehicles become lighter, even percentual weight reductions can represent important savings in battery life and fuel efficiency. In this study, there was a 10% weight reduction for foamed door panels. However, weight reduction comes at the cost of decreased mechanical performance. Although it is difficult to draw direct correlations between individual MS variables and the mechanical response due to the complex geometry and mold filling pattern, it is useful to look at generalized properties to assess the impact of MIM. Figure 26 presents the comparison of specific properties of the foamed part with respect to the compact one. Except for absorbed energy, the compact material still performs better per unit of mass. Yet, studies have shown it is possible to increase some specific properties by using MIM [85,86]. Optimizing the performance for foamed LGF composites is no trivial task. Therefore, design and material engineers need to include the interaction between fiber and foam MS in their design consideration during component development. Modeling tools have improved to the point where both microstructural variables predictions are fairly accurate. Nevertheless, a strong understanding of the underlying physics is necessary to make the right decisions during the simulation process.

## Figures and Tables

**Table 1:** Sources of vibration. Frequency value for (*) a light truck equipped with automatic transmission, 787 mm tires, traveling at 80 km/h in overdrive, and (°) assuming a maximum engine speed of 6000 rpm.

Vibration Source	Frequency [Hz]	Reference
Suspension and wheel	~10	[31,32]
Engine*°	<100	[31]
Body	25–40	[32]
Driveline	50–150	[31,32]
Road defects	<200	[30,32,33,34]
Door slam	<170	[35]

**Table 2:** Processing conditions for the IM trials.

Setting	Compact	Foamed
Nitrogen Gas pressure [MPa]	0	2
Melt Temperature [°C]	245	245
Mold Temperature [°C]	50	85
Back Pressure [MPa]	11	8
Injection Pressure [MPa]	90	120
Holding Pressure [MPa]	27	27
Holding Time [s]	15	0

**Table 3:** Fiber orientation tensors for compact and foamed panels.

	Compact Panels			Foamed Panels		
Location	A_xx_	A_yy_	A_zz_	A_xx_	A_yy_	A_zz_
1 Weld line	0.22	0.75	0.03	0.28	0.68	0.04
2 Gate	0.39	0.56	0.05	0.46	0.50	0.05
3	0.60	0.35	0.05	0.64	0.32	0.04
4	0.50	0.43	0.07	0.53	0.44	0.03
5	0.52	0.40	0.08	0.59	0.37	0.04
6 Rib	0.05	0.26	0.69	0.04	0.19	0.77

**Table 4:** Representative values for foamed and compact samples from drop-tower impact testing.

Specimen	Normalized Impact Force [kN/kg]	Normalized Equivalent Impact Stiffness [kN/mm/kg]	Normalized Absorbed Energy [J/kg]
compact	13.25	2.19	141.01
foamed	13.01 ± 0.25	1.86 ± 0.003	187.63 ± 1.07

**Table 5:** Transverse crack propagation length for compact and foamed specimen.

Specimen	Crack Propagation Length [mm]	
	Impact Side	Back Side
compact	15.15	21.10
foamed	7.58 ± 1.50	13.17 ± 1.45

**Table 6:** Estimated natural frequencies, mode descriptions, and damping ratios for the first nine modes of door panels.

	Frequency [Hz]		Damping Ratio [%]		Mode Description
	Compact	Foamed	Compact	Foamed	
Mode 1	36.73	36.37	2.95	1.99	Plate Mode 1
Mode 2	38.06	37.79	2.15	2.72	Bending along y-axis
Mode 3	73.65	72.67	1.98	1.92	Plate Mode 2
Mode 4	89.20	88.04	1.87	1.78	Bending along x-axis
Mode 5	127.62	124.67	2.44	2.64	Plate Mode 3
Mode 6	136.21	133.38	2.19	2.41	Plate Mode 4
Mode 7	142.36	141.03	2.11	2.00	Bending along y-axis
Mode 8	160.11	160.30	2.10	2.01	Bending along x-axis
Mode 9	168.60	170.90	2.15	2.00	Bending along y-axis

**Table 7:** Estimated natural frequencies, mode descriptions, and damping ratios of tested composite beams. All bending modes are along the y-axis.

	Frequency [Hz]		Damping Ratio [%]		Mode Description
	Compact	Foamed	Compact	Foamed	
Mode 1	72.98	69.41	2.80	3.01	Bending Mode 1
Mode 2	210.65	198.50	3.44	3.37	Bending Mode 2
Mode 3	395.70	370.83	5.94	6.51	Bending Mode 3

**Table 8:** Estimated specific elastic moduli.

	Compact [m^2^/s^2^] × 10^6^	Foamed [m^2^/s^2^] × 10^6^
Mode 1	3.58	3.36
Mode 2	3.93	3.61
Mode 3	3.61	3.28

## Data Availability

The data presented in this study are available upon request from the corresponding author.
